# SDA-SwinNet: Swin-UNet with Dense Skip and Shift-ASPP for Retinal Vessel Segmentation

**DOI:** 10.3390/s26154899

**Published:** 2026-08-03

**Authors:** Jinghua Xiao, Ming Zhao, Rui Yang, Zhengqiang Wang, Tie Luo, Wangyu Wu

**Affiliations:** 1 School of Computer Science, Yangtze University, Jingzhou 434025, China; 2024720821@yangtzeu.edu.cn (J.X.); hitmzhao@gmail.com (M.Z.); 201900912@yangtzeu.edu.cn (R.Y.); 2 School of Cyber Science and Engineering, Wuxi University, Wuxi 214105, China; luotieh@gmail.com; 3 Wuxi Key Laboratory of Artificial Intelligence and Security, Wuxi University, Wuxi 214105, China; 4 School of Physics & Electronic Engineering, Hubei University of Arts and Science, Xiangyang 441053, China; 5 School of Computer Science, University of Liverpool, Liverpool L69 3DR, UK

**Keywords:** fundus vessel segmentation, ASPP, Swin-UNet, deep learning, medical image analysis, Swin transformer encoder

## Abstract

Retinal artery/vein segmentation is a prerequisite for many ophthalmic diagnostic tools. Yet, the task remains difficult: vessels form complex trees, vary widely in caliber, and often appear low-contrast at terminal branches. We propose SDA-SwinNet to handle these challenges. The network adopts Swin-UNet as its backbone and adds three modifications: a Shift-ASPP module for multi-scale context, an HF-Bridge for cross-level feature fusion, and a fractal-constrained loss with a differentiable topology surrogate. Experimental results on the DRIVE-AV and LES-AV datasets show that the proposed model achieves an overall F1-score of 73.13% on DRIVE-AV and 67.85% on LES-AV, with additional class-wise evaluations for arteries and veins. The results demonstrate that SDA-SwinNet achieves a competitive trade-off between segmentation accuracy and computational efficiency.

## 1. Introduction

Retinal vessels play an important role in the assessment of ocular and systemic diseases. Abnormal retinal vascular morphology, such as changes in vessel width, tortuosity, branching structure, and vascular density, can provide valuable information for disease screening and clinical analysis. For example, retinal vessel morphology has been investigated in studies of diabetic retinopathy, glaucoma-related risk assessment, and vascular feature measurement from fundus images [1,2,3,4]. Accurate vessel segmentation is therefore a fundamental step for subsequent quantitative analysis of retinal vascular structures.

Retinal vessel segmentation has long been studied in medical image analysis. Early studies relied mainly on hand-crafted features, ridge detection, ensemble classifiers, and structured prediction methods to extract vessel pixels from color fundus images [3,5,6]. Public datasets such as DRIVE and RITE have also promoted the development and evaluation of retinal vessel segmentation and artery–vein classification algorithms [3,7]. However, traditional methods are often sensitive to illumination variation, low contrast, vessel thickness changes, and pathological interference.

With the development of deep learning, convolutional neural networks have significantly improved the performance of medical image segmentation, including retinal vessel segmentation [8,9]. Fully convolutional networks provide an end-to-end framework for semantic segmentation [10], while U-Net introduces an encoder–decoder structure with skip connections and has become a widely used baseline in biomedical image segmentation [11]. Based on U-Net, many improved architectures have been proposed to enhance feature extraction and segmentation accuracy, such as UNet++ [12], ResUNet++ [13], CE-Net [14], DUNet [15], SA-UNet [16], and other retinal vessel segmentation networks [17,18,19].

Recent studies have further explored multi-scale feature extraction, attention mechanisms, deformable convolutions, topology-preserving constraints, and clinically relevant loss functions to improve the segmentation of thin vessels and complex vascular structures [15,16,20,21,22,23]. These methods aim to address common challenges in retinal vessel segmentation, including discontinuous thin vessels, blurred boundaries, class imbalance, and the preservation of vascular topology. Therefore, improving the accuracy and robustness of retinal vessel segmentation remains an important research direction in medical image analysis.

Recent studies have improved retinal vessel segmentation by incorporating additional structural constraints during network training. For example, topology-preserving loss functions and structure-aware constraints have been designed to guide networks toward learning more continuous vascular structures, thereby alleviating vessel discontinuity and improving the preservation of thin tubular regions [24,25]. In addition, clinically relevant, feature-optimized losses have also been explored for retinal vessel segmentation and vascular feature measurement [23].

Despite these advances, existing methods still face multiple challenges in real clinical scenarios. On the one hand, retinal vessels exhibit complex branching patterns and strong multi-scale characteristics. In particular, fine vessels, terminal regions, and artery–vein crossing areas are often associated with low contrast, local occlusion, and blurred boundaries, leading to unstable segmentation in these regions. On the other hand, conventional convolutional neural networks are limited in modeling long-range dependencies and global structural consistency, whereas Transformer-based models have stronger global modeling ability but may still require effective mechanisms to enhance local texture representation and boundary detail preservation [26,27,28].

Meanwhile, retinal vascular structures show tree-like and self-similar morphological characteristics. However, most current methods mainly rely on pixel-level or region-level objectives, such as cross-entropy loss and Dice-based losses, and do not sufficiently exploit higher-order morphological priors of vascular structures. As a result, the generated vessel maps may suffer from fragmentation, topological inconsistency, or over-smoothing. Incorporating vascular morphological priors into the loss function is therefore a promising direction for improving structural consistency and segmentation quality [21,22,23].

In addition, many existing networks remain insufficient at capturing multi-scale information when dealing with slender, tree-like, and multi-scale retinal vessels. Although atrous spatial pyramid pooling (ASPP) and dilated convolution have been widely used to enlarge the receptive field and aggregate multi-scale contextual information [29,30,31,32,33], dilated convolution may introduce gridding artifacts, particularly in small-vessel regions. Furthermore, the semantic gap between shallow detail features and deep semantic features may cause the loss of fine vascular structures during feature fusion.

Motivated by the above issues, this paper proposes a high-precision and structure-sensitive retinal vessel segmentation model. Using Swin-UNet as the backbone [27], the proposed model introduces an improved Shift-ASPP module to expand the receptive field with low computational cost and better capture multi-scale vascular structures [33,34]. In addition, a hierarchical feature bridge module, namely HF-Bridge, is designed to enhance the fusion of cross-level semantic and detail features. Finally, a fractal-constrained loss is proposed to incorporate the self-similar morphological characteristics of retinal vessels, thereby improving structural complexity preservation and topological consistency. Experimental results on multiple public datasets demonstrate that the proposed model achieves superior segmentation performance compared with existing methods. The main contributions are summarized as follows:

(1) Swin-UNet-based backbone design: The proposed network adopts a Swin-UNet-like encoder–decoder architecture and enhances it with lightweight multi-scale feature modeling and cross-level feature fusion, aiming to improve artery–vein segmentation accuracy while maintaining practical inference efficiency.

(2) We adopt CF-Loss from [24] as a structural regularizer and further combine it with a differentiable topology-aware surrogate to enforce vascular connectivity.

(3) Improved ASPP and hierarchical feature bridge module: We introduce a shift operation into ASPP to expand the receptive field at zero cost and alleviate the gridding effect. Combined with the HF-Bridge, this design enhances contextual modeling and feature fusion while preserving vascular details.

## 2. Related Works

### 2.1. Swin-UNet

Since U-Net was proposed by Ronneberger et al. [11], its symmetric encoder–decoder architecture and skip-connection design have made it a landmark model in medical image segmentation. By integrating shallow high-resolution features with deep semantic features, U-Net provides an effective framework for segmenting fine structures and has been widely adopted in retinal vessel segmentation [8,9,11]. However, the original U-Net still has limitations in representing complex vascular morphology, especially in thin vessels, blurred boundaries, and low-contrast regions.

To overcome these limitations, numerous U-Net variants have been proposed to improve feature representation, contextual modeling, and boundary preservation. For example, UNet++ introduces nested and dense skip connections to reduce the semantic gap between encoder and decoder features [12]. ResUNet++ combines residual learning with the U-Net framework to enhance feature extraction and improve segmentation robustness [13]. CE-Net further strengthens contextual representation through a context encoder structure [14]. In retinal vessel segmentation, DUNet introduces deformable convolution to better adapt to vessel shape variations [15], while SA-UNet integrates a spatial attention mechanism into U-Net to improve region selectivity and fine-vessel recognition [16].

To address the limited receptive field of conventional convolution kernels, dilated convolutions and pyramid-based structures have been introduced into segmentation networks for multi-scale contextual aggregation [29,30,31,32]. Atrous spatial pyramid pooling can enlarge the receptive field and capture vessels of different calibers, thereby improving the representation of multi-scale vascular structures. However, dilated convolution may also introduce gridding artifacts, especially in small-vessel regions, which can affect the continuity and completeness of fine vascular branches [32,33].

Meanwhile, several studies have attempted to improve structural consistency by introducing structure-aware constraints during network training. Topology-preserving loss functions and skeleton-based losses can guide the network to maintain tubular continuity and reduce vessel breakage [21,22]. Clinically relevant, feature-optimized loss functions have also been proposed to improve vascular feature measurement and multi-class vessel segmentation [23]. These studies show that structural constraints are important for improving the morphological fidelity of retinal vessel segmentation.

With the development of Vision Transformers, their global modeling capacity has been increasingly incorporated into medical image segmentation [26]. Compared with conventional convolutional networks, which mainly rely on local receptive fields, Transformer-based models can capture long-range dependencies through self-attention, making them suitable for modeling tree-like retinal vascular structures with complex spatial relationships. Swin-UNet incorporates Swin Transformer blocks into the U-Net-like encoder–decoder framework, retaining progressive spatial recovery while enhancing global representation capability [27].

In retinal vessel segmentation, Swin-UNet and its variants have shown potential for alleviating fine-vessel fragmentation, incomplete segmentation, and background noise interference. For example, TD-Swin-UNet introduces texture-driven enhancement modules to improve the representation of small vessels, blurred boundaries, and vascular topology [28]. These methods demonstrate that combining global contextual modeling with local texture enhancement is beneficial for retinal vessel segmentation.

Nevertheless, Swin-UNet still faces several challenges in retinal vessel segmentation, including insufficient local detail representation, information loss during cross-level feature transmission, and a weaker texture modeling ability compared with convolutional neural networks. Therefore, how to effectively combine the global modeling ability of Transformers with the local texture representation of CNNs has become an important research direction. Against this background, recent studies have increasingly explored hybrid Transformer–CNN architectures, lightweight multi-scale feature fusion, and topology-constrained loss functions to further improve segmentation accuracy, structural consistency, and clinical usability [27,28,29].

### 2.2. ASPP

Retinal vessels exhibit pronounced multi-scale structural characteristics, including thick arterial and venous trunks, fine peripheral vessels, and highly irregular structures in terminal bifurcation regions. Accordingly, sufficiently rich multi-scale feature extraction is crucial for improving the robustness and structural consistency of vessel segmentation. In this context, atrous convolution and atrous spatial pyramid pooling (ASPP) have been widely used for multi-scale contextual modeling in semantic segmentation and medical image segmentation [29,30,31,32].

ASPP was introduced to expand the receptive field of convolutional neural networks while maintaining the spatial resolution of feature maps. By setting different dilation rates in parallel branches, ASPP can simultaneously capture local details and larger contextual information. Its main advantage is that it enlarges the effective receptive field without additional pooling operations or excessive downsampling, thereby improving the representation of objects and structures at different scales [29,30].

In medical image segmentation, ASPP has been incorporated into several U-Net-based frameworks to enhance multi-scale feature extraction. For example, ResUNet++ combines the U-Net structure with residual learning and ASPP to strengthen contextual representation [13]. U-Net-ASPP introduces atrous spatial pyramid pooling into the U-Net framework to improve medical image segmentation performance [35]. Bridged-U-Net-ASPP further combines ASPP with an optimized U-Net-based structure for brain tumor MRI segmentation [36]. These studies indicate that ASPP can effectively improve contextual aggregation and multi-scale representation in medical image segmentation.

However, conventional ASPP still has limitations when applied to retinal vessel segmentation. Owing to the sparse sampling mechanism of dilated convolution, large dilation rates may introduce gridding artifacts, preventing the model from continuously capturing tiny vessels, edge details, and gradient variations in tortuous vessel regions [32]. In addition, fixed combinations of dilation rates are not sufficiently flexible for handling vessels of different calibers and may fail to fully adapt to the complex tree-like morphology of retinal vessels.

To overcome these issues, researchers have proposed various improvements to multi-scale feature extraction modules. Some methods combine dilated convolution with attention mechanisms to enhance the response to important regions, while others introduce depthwise separable convolution to reduce computational cost [30]. In addition, multi-scale feature aggregation, cross-layer feature fusion, and topology-sensitive loss functions have also been explored to compensate for the limitations of conventional ASPP in structural modeling [21,22,23].

Although these improvements have achieved certain gains, how to further enlarge the effective receptive field while maintaining a lightweight design and alleviating gridding artifacts remains a key challenge when ASPP is applied to fine-vessel segmentation. Recent studies have shown that feature-space shift operations can obtain additional local neighborhood information by spatially shifting feature maps without introducing extra parameters or floating-point operations [34]. This provides a useful direction for improving ASPP in lightweight segmentation networks.

Building on the classical ASPP framework, this paper introduces a Shift-ASPP module that enlarges the receptive field through channel-wise spatial shift and improves multi-scale structural representation. Compared with conventional ASPP, the proposed module is better suited to the slender, multi-scale, and tree-like characteristics of retinal vessels, especially for preserving fine vascular branches and improving structural consistency.

### 2.3. Clinically Relevant Vascular Features

Retinal vascular features, such as vessel tortuosity, vessel density, branching patterns, and other morphology-related measurements, have been widely investigated in retinal image analysis and clinical research [2,4,23]. These features provide quantitative descriptions of vascular morphology and are important for downstream clinical analysis, disease-related assessment, and vascular biomarker measurement.

Among these vascular descriptors, structural complexity is particularly important for retinal vessel analysis. Retinal vessels exhibit tree-like branching patterns, strong self-similarity, and irregular morphology in terminal and peripheral regions. These characteristics make it necessary for segmentation models to preserve not only pixel-level accuracy but also global vascular structure and morphological continuity. However, most existing segmentation networks are mainly optimized using pixel-wise or region-wise losses, such as cross-entropy loss and Dice-based losses, which may be insufficient for preserving higher-order vascular morphology.

Vessel density and other vascular measurements are typically computed from segmentation maps. Therefore, inaccurate segmentation, broken vessels, missing fine branches, or over-smoothed boundaries can directly affect the reliability of subsequent vascular feature estimation. Recent studies have begun to incorporate clinically relevant vascular features into the optimization process, showing that feature-aware constraints can improve segmentation quality and vascular measurement reliability [23].

Nevertheless, many vascular descriptors involve non-differentiable or weakly differentiable operations, making them difficult to directly optimize in conventional end-to-end segmentation networks. To address this limitation, this paper introduces a fractal-constrained loss that incorporates the self-similar and tree-like characteristics of retinal vessels into network training. By encouraging the model to preserve structural complexity and vascular continuity, the proposed loss aims to improve both segmentation accuracy and the reliability of downstream vascular feature estimation.

## 3. Methods

### 3.1. Problem Formulation and Overall Optimization Objective

The retinal artery–vein segmentation task can be formulated as a multi-class segmentation problem with additional structural constraints. Given an input fundus image, the goal is to predict artery, vein, and background probability maps. Pixel-level supervision is applied to the artery and vein classes, while structural constraints are computed on the merged vessel foreground map to preserve the morphology and connectivity of the complete vascular tree:

(1) $${min}_{\theta }L_{\mathrm{total}}=E_{\mathrm{data}}+\beta E_{\mathrm{fractal}}+\gamma E_{\mathrm{topology}}$$

Each energy term is defined as follows:

$E_{\mathrm{data}}$ ensures pixel-level prediction accuracy.

$E_{\mathrm{fractal}}$ maintains the intrinsic complexity of the vascular network.

$E_{\mathrm{topology}}$ preserves vascular connectivity.

### 3.2. Unified Formulation of Multi-Scale Feature Learning

The network constructs a hierarchical feature manifold and progressively extracts multi-scale representations through a Swin Transformer backbone:

(2) $$F=\{F_{0},F_{1},F_{2},F_{3},F_{4}\}$$

Each feature level is generated through successive transformations:

(3) $$\begin{array}{cc} & F_{0}=\Psi_{\mathrm{patch}}\left(I\right)\\ F_{i+1} & =\Psi_{\mathrm{Swin}}^{\left(i\right)}(F_{i})=\mathrm{MLP}(\mathrm{LN}(\mathrm{W-MSA}(\mathrm{LN}(F_{i}))))+F_{i},i=0,\ldots ,3 \end{array}$$

Here, LN denotes layer normalization, W-MSA denotes window-based multi-head self-attention, and MLP denotes a multi-layer perceptron.

### 3.3. Mathematical Foundations of Structural Constraints

#### 3.3.1. Fractal Geometric Constraint

The retinal vascular network exhibits pronounced self-similarity in a statistical sense and can be modeled as a statistical fractal. Let the binary vessel mask be denoted as V; its fractal dimension is defined via the box-counting method as follows:

(4) $$\mathrm{FD}(V)={lim}_{\epsilon \to 0}\frac{logN(\epsilon )}{log(1/\epsilon )}$$

where N(ϵ) denotes the minimum number of boxes with side length ϵ required for coverage.

We further introduce a multi-scale fractal descriptor to characterize the scale-dependent structural complexity of the vasculature:

(5) $$\mathrm{MFD}(V)=\{{\mathrm{FD}}_{\sigma_{1}}(V),{\mathrm{FD}}_{\sigma_{2}}(V),\ldots ,{\mathrm{FD}}_{\sigma_{n}}(V)\}$$

(6) $$E_{\mathrm{fractal}}=|\mathrm{FD}(\hat{Y})-\mathrm{FD}(Y)|+\lambda \sum_{\sigma \in \Sigma }∥{\mathrm{FD}}_{\sigma }(\hat{Y})-{\mathrm{FD}}_{\sigma }(Y){∥}_{2}$$

The multi-scale fractal descriptor MFD(V) represents the fractal complexity of the vascular structure at different scales. Instead of relying only on a single global fractal dimension, it computes scale-dependent fractal dimensions under a set of scales Σ. The fractal energy term $E_{fractal}$ penalizes the discrepancy between the predicted vessel mask $Y^{\hat{Y}}$ and the ground-truth mask Y in both global and multi-scale fractal dimensions. The first term enforces global morphological complexity consistency, while the second term constrains scale-dependent structural similarity, thereby encouraging the model to preserve fine vessels, branching patterns, and overall vascular complexity.

#### 3.3.2. Topology-Preserving Constraint

The topological properties of the vascular network are considered from the perspective of connectivity and loop consistency. In theory, persistent homology can describe connected components and holes, but exact computation involves thresholding and discrete topological matching, which are not differentiable. Therefore, in the actual implementation, we use a differentiable topology-aware surrogate based on soft skeleton consistency to encourage vascular connectivity during training. The Betti error, which measures the difference in connected components and holes between the prediction and ground truth, is computed only after inference as an evaluation metric.

### 3.4. Variational Interpretation of the Network Architecture

The proposed SDA-SwinNet can be regarded as a parameterized solver for the above variational problem. Each module in the network corresponds explicitly to a term in the optimization objective.

The Swin Transformer encoder constructs the feature manifold and provides multi-level feature representations for the data fidelity term.

The Shift-ASPP module explicitly optimizes multi-scale perception and corresponds to the boundary-aware component in the energy functional.

The HF-Bridge module promotes feature fusion through dense cross-layer connections and supports the modeling of complex structures.

CFLoss approximates the fractal and topology-aware structural constraint terms through differentiable soft box-counting and soft connectivity supervision.

This unified mathematical framework not only provides theoretical guidance for method design but also lays a solid foundation for subsequent generalizations and extensions.

### 3.5. Overall Framework

**Figure 1:**
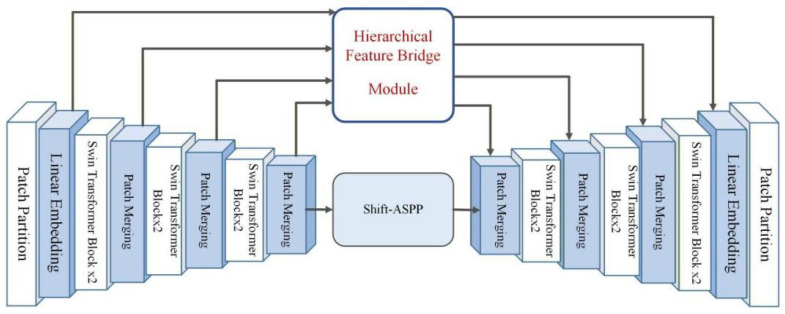
Overall architecture diagram of the model.

The proposed network is built upon the Swin Transformer and combines multi-scale feature enhancement with cross-layer feature fusion to achieve high-accuracy retinal vessel segmentation with relatively high parameter efficiency. The overall architecture adopts a U-shaped, symmetric encoder–decoder design and consists of four principal components: a Swin Transformer encoder, an improved Shift-ASPP multi-scale feature enhancement module, an HF-Bridge hierarchical feature bridging module, and a Swin Transformer decoder. A schematic illustration of the architecture is shown in Figure 1.

In the encoding stage, the input fundus image is first divided into a series of non-overlapping patches by the Patch Embedding operation, and projected into a high-dimensional feature space. The features are then sequentially fed into an encoder composed of multiple Swin Transformer blocks. By alternately employing window-based multi-head self-attention (W-MSA) and shifted-window multi-head self-attention (SW-MSA), the encoder effectively models local contextual information and long-range cross-window dependencies while controlling computational complexity, thereby enabling hierarchical modeling of global vessel trajectories and topology.

The multi-scale features output by the encoder are further processed at the bottleneck layer. To enhance the network’s perception of vessel structures at different scales, an improved Shift-ASPP module is introduced at this stage. The module applies a spatial shift to channel-wise features before convolution, expanding the effective receptive field without introducing additional learnable parameters and alleviating the gridding effect that may arise in conventional dilated convolution, thereby improving the response to fine vessels and complex branching regions.

The decoder uses a standard Swin Transformer structure symmetric to the encoder. Between the encoder and decoder, we insert HF-Bridge—a module that fuses shallow and deep features across scales. This is not a new idea per se, but our implementation uses Res2Net blocks within the bridge to better preserve multi-scale information. We found that this reduces the semantic gap that often causes fine vessels to disappear during upsampling.

By fully integrating the global modeling ability of Swin Transformer, the multi-scale enhancement of Shift-ASPP, the cross-layer fusion of HF-Bridge, and the structural constraints of CFLoss, the overall network achieves highly accurate and highly robust segmentation of retinal vascular structures.

### 3.6. Shift-ASPP

To expand the effective receptive field of ASPP without increasing the number of parameters or computation, we first apply a channel-wise spatial shift operation to the inputs of the parallel ASPP branches. Specifically, we divide the input feature channels into eight groups and shift each group by one pixel (s=1) in one of the eight directions (up, down, left, right, and the four diagonals). For pixels that are shifted beyond the original feature map boundary, zero padding is applied, i.e., the newly introduced positions are filled with zeros. This padding strategy introduces no extra learnable parameters and keeps the receptive field expansion mathematically tractable. Under the assumption that the feature map size is sufficiently larger than 2s, the boundary effect caused by zero padding is negligible, and the effective receptive field of a subsequent dilated convolution can be approximated as 1+2d+2s in each dimension.

For a single 3 × 3 dilated convolution with dilation rate d, the effective width covered along each axis (i.e., the receptive-field length, in pixels) is given by

(7) $$R_{\mathrm{conv}}\left(d\right)=1+2d$$

Because the spacing between adjacent sampling points in a 3 × 3 kernel is d, the radius from the center to the farthest sampling point is d, and the total width on both sides is therefore 1 + 2d. When ASPP uses several dilation rates in parallel, the receptive field of each branch is given by the above expression, and the maximum receptive field of the overall parallel structure is the maximum among all branches:

(8) $$R_{\mathrm{ASPP}}=\underset{k}{max}\left(1+2d_{k}\right)$$

Under the standard ASPP setting with d = {6, 12, 18}, the receptive fields of the individual branches are as follows:

(9) 1+2⋅6=13, 1+2⋅12=25, 1+2⋅18=37

The role of shift is to spatially translate part of the input channels so that the subsequent convolution can access information outside the original receptive field. If the maximum shift magnitude is s pixels, then for any subsequent convolution branch, the receptive-field radius in each spatial direction increases by s, and the receptive-field length therefore increases by 2 s. Accordingly, for a single 3 × 3 dilated branch, the effective receptive field after introducing shift becomes

(10) $$R\prime_{conv}\left(d,s\right)=1+2d+2s\;(with\;zero\;padding)$$

The derivation assumes that the zero-padding region does not significantly distort feature statistics when s is small (e.g., s=1) and the feature map resolution is high (e.g., ≥64 × 64). In our experiments with 720 × 720 input resolution, this condition is satisfied.

For the ASPP module as a whole, the maximum receptive field after introducing shift is as follows:

(11) $$R\prime_{\mathrm{ASPP}}=\underset{k}{max}\left(\begin{array}{c} 1+2d_{k}+2s \end{array}\right)$$

Applying a spatial offset of at most s pixels to part of the channels before convolution is equivalent to increasing the receptive field of each ASPP/dilated branch by 2 s in each dimension, i.e., increasing the side length by 2 s.

Taking the common ASPP dilation rates d = {6, 12, 18} (corresponding to receptive fields of 13, 25, and 37) and setting the shift step to s = 1, the area increases from 132 = 169 to 152 = 225, an improvement of approximately 33.1%; from 625 to 729, an improvement of approximately 16.6%; and from 1369 to 1521, an improvement of approximately 11.1%. These results indicate that branches with smaller dilation rates benefit more noticeably in both absolute and relative area gain, although all branches obtain additional local neighborhood information, which is beneficial for capturing terminal and fine vessels.

**Figure 2:**
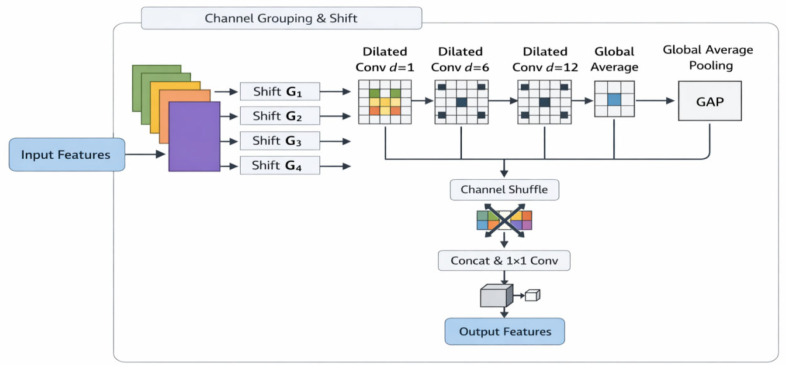
Structural schematic of the Shift-ASPP module.

Figure 2 illustrates the structure of the Shift-ASPP module. The Shift-ASPP module can be interpreted as introducing structured prior constraints into a multi-scale feature space, and its mathematical form corresponds to adding a multi-scale regularization term to the variational problem.

Given the input feature map X, we first divide it into K channel groups $\{X_{k}{\}}_{k=1}^{K}$. For the k−th group, a spatial shift operation with offset (Δxk,Δyk) is applied to introduce directional neighborhood information without additional learnable parameters. The shifted feature is then processed by a dilated convolution with dilation rate $d_{k}$, producing the branch output $T_{k}(X)$. Different branches correspond to different shift directions and dilation rates, allowing the module to capture vessel structures under multiple spatial scales and orientations. Finally, all branch outputs are concatenated along the channel dimension and fused by a 1×1 convolution to obtain the multi-scale representation Y:

(12) $$T_{k}(X)={\mathrm{Conv}}_{d_{k}}\left({\mathrm{Shift}}_{\Delta x_{k},\Delta y_{k}}(X^{\left(k\right)})\right),k=1,\ldots ,K$$

The fusion of all branch outputs can be written as

(13) $$Y=F\left(\{T_{k}(X){\}}_{k=1}^{K}\right),F={\mathrm{Conv}}_{1\times 1}\mathrm{Concat}\;$$

Variational correspondence: Shift-ASPP is equivalent to introducing a multi-scale structural consistency term into the energy function:

(14) $$R_{\mathrm{multi-scale}}(Y)=\sum_{k=1}^{K}\lambda_{k}∥\nabla_{d_{k}}Y-T_{k}(X){∥}^{2}$$

The multi-scale regularization term encourages the fused feature representation Y to preserve the structural information extracted by each Shift-ASPP branch. Specifically, $T_{k}(X)$ denotes the feature representation generated by the k-th branch with the dilation rate $d_{k}$, while $\nabla_{d_{k}}Y$ represents the structural gradient of the fused output at the corresponding scale. By minimizing the discrepancy between $\nabla_{d_{k}}Y$ and $T_{k}(X)$, the network is encouraged to maintain consistency between the final fused representation and the scale-specific structural priors learned from different branches. The coefficient $\lambda_{k}$ controls the contribution of each scale to the overall regularization.

Therefore, by explicitly introducing multi-scale convolution kernels and spatial shifts, the Shift-ASPP module compels the network to preserve vascular structural consistency across multiple scales, directly corresponding to the structural regularization term in the energy-minimization framework.

In general, if the ASPP branches contain a dilation-rate set {$d_{k}$} and the maximum shift step is s pixels, then the receptive field of branch k in the improved ASPP is

(15) $$R\prime_{k}=1+2d_{k}+2s$$

The maximum receptive field of the entire module is

(16) $$R\prime_{ASPP}=\underset{k}{max}R\prime_{k}=\underset{k}{max}\left(1+2d_{k}\right)+2s=R_{ASPP}+2s$$

The input feature first enters the feature-tensor representation on the left side of the module. Subsequently, a portion of the channels is selected and fed into the directional shift unit, which performs zero-cost spatial displacements in eight directions to simulate a broader spatial sampling range. The shifted features and the remaining unshifted channels together form a “partially shifted channel” structure, thereby expanding the effective receptive field without increasing the computational burden.

The shifted features are then sent to multiple convolution branches at different scales (represented in the figure by several parallel elongated blocks), corresponding to the common multi-dilation convolutions in ASPP (e.g., 1 × 1, 3 × 3 with dilation 6, dilation 12, and dilation 18). By pre-diffusing the local receptive domain through the shift operation, the multi-scale convolution branches can capture global contextual information and fine-grained vascular structures more effectively.

The features generated by the multi-scale branches are then jointly fed into a Channel Shuffle module on the right. By reordering channels across groups, this module enhances feature interaction among different branches, allowing multi-scale features and direction-shift features to be mixed more thoroughly and improving the overall representational capacity while reducing redundant channel dependence.

### 3.7. HF-Bridge

To effectively alleviate the semantic gap between the encoder and decoder and improve the network’s description of multi-scale vascular structures, we propose the HF-Bridge (hierarchical feature bridge module). Taking the multi-level feature output by the Swin Transformer encoder as the input, the module performs hierarchical alignment, progressive fusion, and multi-scale convolutional enhancement, thereby integrating shallow textures, deep semantics, and cross-scale structural information into a unified representation and improving overall vessel segmentation performance.

Retinal vessels exhibit evident multi-scale, tree-like, and topologically complex characteristics. Conventional skip connections, such as those in U-Net, directly pass shallow features of the same scale to the decoder and therefore cannot adequately integrate semantic information from different depths. This leads to several deficiencies: discontinuous fine vessels, obvious fragmentation, ineffective transmission of deep semantic information to the shallow reconstruction path, and a lack of unified modeling for vessels at multiple scales. To address these problems, HF-Bridge employs dense cross-layer connections, upsampling alignment, and Res2Net-based multi-scale convolutional fusion so that every feature level can acquire complementary information from structures of different depths and scales, yielding feature representations with stronger structural consistency and higher resolution.

HF-Bridge takes the multi-scale outputs of the encoder as the input. Suppose that the features output by the encoder at different depths are denoted by $\left\{F_{0},F_{1},F_{2},F_{3},F_{4}\right\}$, where denotes the spatial feature at layer; the deeper the layer, the lower the spatial resolution, namely,

(17) $$H_{0}>H_{1}>H_{2}>H_{3}>H_{4}$$

To realize cross-scale feature fusion, HF-Bridge adopts a progressive construction strategy. First, high-level features are upsampled to the target scale via bilinear interpolation so that they are spatially aligned with low-level features:

(18) $${\hat{F}}_{i}=\mathrm{Up}\left(F_{i}\right),\;{\hat{F}}_{i}\in R^{B\times H_{0}\times W_{0}\times C_{i}}$$

where $\mathrm{Up}\left(F_{i}\right)$ denotes the upsampling operation.

(1) Primary Bridge Fusion concatenates the shallowest layer with its adjacent deeper feature: $Z_{0,1}=\mathrm{Concat}\left(F_{0},{\hat{F}}_{1}\right)$. A Res2NetBlock is then used to generate multi-scale enhanced features: $X_{0,1}=\mathrm{Res}2\mathrm{Net}\left(Z_{0,1}\right)$. Similarly, the same operation is applied to the features at levels 1 and 2:

(19) $$Z_{1,1}=Concat\left(F_{1},{\hat{F}}_{2}\right),\;X_{1,1}=Res2Net\left(Z_{1,1}\right)$$

(2) Intermediate Bridge Fusion further integrates deeper features so that multi-level semantics are progressively aggregated toward shallow layers:

(20) $$Z_{0,2}=Concat\left(F_{0},X_{0,1},Up\left(X_{1,1}\right)\right)$$

The fused features are then processed by a Res2NetBlock to extract multi-scale representations: $X_{0,2}=Res2Net\left(Z_{0,2}\right)$. Analogously, we obtain

(21) $$Z_{1,2}=Concat\left(F_{1},X_{1,1},Up\left(X_{2,1}\right)\right)X_{1,2}=Res2Net\left(Z_{1,2}\right)$$

(3) Final Bridge Fusion. Finally, HF-Bridge aggregates all hierarchical fusion results at the spatial size of level 0, thereby constructing the final high-resolution multi-scale representation:

(22) $$Z_{0,3}=Concat\left(F_{0},X_{0,1},X_{0,2},Up\left(X_{1,2}\right)\right)$$

A subsequent Res2NetBlock then produces the final hierarchically fused feature: $X_{0,3}=Res2Net\left(Z_{0,3}\right)$, where denotes the highest-resolution feature output by HF-Bridge.

(4) Transformer-Friendly Feature Reprojection. To adapt the fused spatial features back to the Transformer architecture, HF-Bridge flattens and restacks them into sequence form:

(23) $${\tilde{X}}_{k}=Flatten\left(X_{k}\right)\in R^{B\times \left(H_{k}W_{k}\right)\times C_{k}},\;k\in \{0,1,2\}$$

The final output sequence set is

(24) $$X_{bridge}=\{{\tilde{X}}_{0},{\tilde{X}}_{1},{\tilde{X}}_{2}\}$$

**Figure 3:**
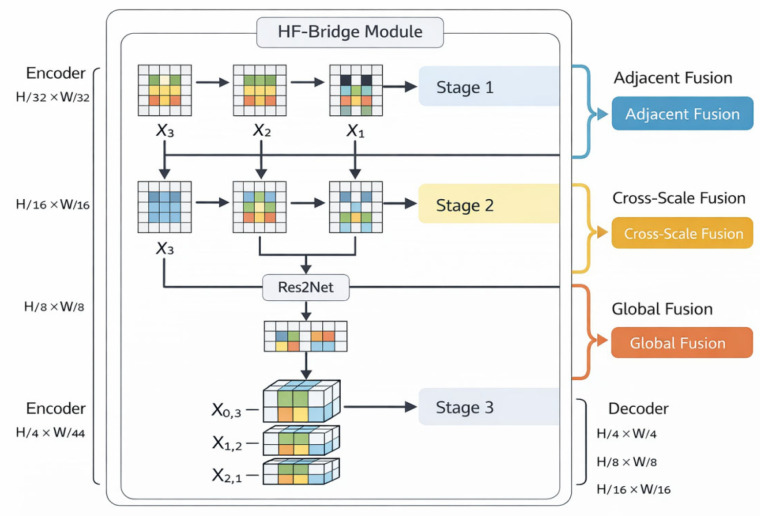
Structural schematic of the HF-Bridge module.

The architecture of HF-Bridge is presented in Figure 3. By densely fusing features from different depths in three stages, HF-Bridge enables the model to attend simultaneously to coarse vessel trajectories and fine capillary structures. The layer-by-layer bridging tightly aligns shallow texture features with deep semantic features and effectively improves segmentation consistency. During fusion, Res2NetBlock provides independent representation paths for grouped convolutions at different scales, significantly improving recognition of fine vessels, bifurcations, and terminal regions. Its output is structurally compact, parameter-efficient, and consistent with the input format required by the Swin Transformer decoder.

Through its distinctive strategy of hierarchical alignment, cross-layer fusion, and multi-scale enhancement, HF-Bridge provides the network with strong structural modeling capabilities. Combined with the global feature representation ability of the Swin Transformer, this module substantially improves the recovery accuracy of fine structures and complex morphology in retinal vessel segmentation.

### 3.8. Loss Function

Our loss function builds upon the CF-Loss framework recently proposed by Zhou et al. [23], which introduces clinically relevant feature optimization for retinal vessel segmentation. We adopt the fractal-dimension-based structural constraints from [23] and combine them with a differentiable topology-aware surrogate to further preserve vascular connectivity. Importantly, the primary novelty of our work lies in the network architecture (Shift-ASPP and HF-Bridge modules), while the loss function serves as a complementary structural regularizer.

To jointly improve class-wise prediction accuracy and structural consistency in retinal artery–vein segmentation, we designed a hybrid loss function composed of pixel-level multi-class segmentation loss, fractal structure constraints, a differentiable topology-aware surrogate, and an optional boundary loss. The overall objective is defined as follows:

(25) $$L_{\mathrm{total}}=L_{\mathrm{pixel}}+\lambda_{1}L_{\mathrm{fractal}}+\lambda_{2}L_{\mathrm{topology}}+\lambda_{3}L_{\mathrm{boundary}\;}$$

The weights of the fractal and topology-aware terms are set to 0.1 and 0.05, respectively. The boundary-loss weight is set to 0 in the main experiments because the boundary loss is disabled, and it is considered only in the ablation analysis.

#### 3.8.1. Pixel-Level Loss

Important Note: The core fractal constraint CF-Loss is adopted from [23], and our novelty lies in the network architecture (Shift-ASPP and HF-Bridge), rather than the loss function itself. We only supplement a differentiable topology surrogate to optimize vascular connectivity.

The pixel-level loss ensures correct class-wise prediction for each pixel. Since the artery–vein task contains artery, vein, and background classes, we use a multi-class pixel-level loss composed of categorical cross-entropy and class-wise Dice losses. The categorical cross-entropy term provides stable class-wise supervision, while the class-wise Dice term directly optimizes the overlap between prediction and ground truth, which is important for handling the imbalance among artery, vein, and background pixels:

(26) $$L_{BCE}=-\frac{1}{N}\sum_{i=1}^{N}\left[y_{i}\log({\hat{y}}_{i})+(1-y_{i})\log(1-{\hat{y}}_{i})\right]$$

(27) $$L_{Dice}=1-\frac{2\sum_{i=1}^{N}y_{i}{\hat{y}}_{i}+\epsilon }{\sum_{i=1}^{N}y_{i}+\sum_{i=1}^{N}{\hat{y}}_{i}+\epsilon }$$

The overall pixel-level loss is as follows:

(28) $$L_{\mathrm{pixel}}=L_{\mathrm{BCE}}+L_{\mathrm{Dice}}$$

The pixel-level loss is designed to ensure accurate classification of each pixel. We combine binary cross-entropy (BCE) loss and Dice loss. The BCE loss penalizes pixel-wise classification errors and provides stable supervision for foreground and background pixels. The Dice loss directly optimizes the overlap between the predicted vessel probability map and the ground-truth mask, which is particularly useful for retinal vessel segmentation due to the severe class imbalance between vessel and background pixels. Therefore, the combined pixel-level loss encourages both accurate pixel-wise prediction and high region-level overlap.

#### 3.8.2. Fractal Structure Constraint

Retinal vascular networks exhibit statistical self-similarity, and the fractal dimension (FD) quantifies their morphological complexity. We use a differentiable soft box-counting approximation to estimate the global fractal descriptor from the predicted vessel probability map:

(29) $$\mathrm{FD}(V)=\underset{\epsilon \to 0}{lim}\frac{logN(\epsilon )}{log(1/\epsilon )}$$

where N(ϵ) is the minimum number of boxes that side length  ϵ needs to cover the vessel pixels. To enforce similarity in global fractal properties between the prediction and the ground truth, we define the following:

(30) $$L_{\mathrm{FD-global}}=|\mathrm{FD}(\hat{Y})-\mathrm{FD}(Y)|$$

However, a single global FD cannot capture the local heterogeneity of the vasculature. We therefore introduce local fractal-dimension distribution alignment. The image is divided into overlapping or non-overlapping patches (a sliding window of size 32 × 32 with stride 8 in our implementation). For each patch, we compute its local FD, forming empirical distributions $P_{\hat{V}}$ for the prediction and $P_{V}$ for the ground truth. Two distance measures are employed.

KL divergence loss is calculated as follows:

(31) $$L_{\mathrm{FD-KL}}=D_{\mathrm{KL}}(P_{\hat{V}}∥P_{V})=\sum_{x\in X}P_{\hat{V}}(x)log\frac{P_{\hat{V}}(x)}{P_{V}(x)}$$

Wasserstein distance loss (approximated by the Sinkhorn algorithm to remain differentiable) is calculated as follows:

(32) $$L_{\mathrm{FD-W}}=W_{p}\left(P_{\hat{V}},P_{V}\right)={\left(\underset{\gamma \in \Gamma \left(P_{\hat{V}},P_{V}\right)}{\inf}\int_{X\times X}|x-y{|}^{p}d\gamma \left(x,y\right)\right)}^{\frac{1}{p}}$$

By combining global and local constraints, the final fractal loss becomes

(33) $$L_{\mathrm{fractal}}=L_{\mathrm{FD-global}}+\alpha L_{\mathrm{FD-KL}}+\beta L_{\mathrm{FD-W}}$$

In our experiments, we set α=0.5 and β=0.5.

#### 3.8.3. Topology Constraint

To preserve the connectivity and completeness of the vascular network, we introduce a differentiable, topology-aware surrogate based on soft skeleton consistency.

Let ${\mathrm{PH}}_{k}(\hat{Y})$ and ${\mathrm{PH}}_{k}(Y)$ denote the persistence diagrams (in dimension k=0 for connected components and k=1 for loops) of the prediction and the ground truth, respectively. Each point in the diagram is a birth–death pair $\left(b_{i},d_{i}\right)$. In the training stage, exact persistence-diagram matching is not directly optimized. Instead, a soft-skeleton-based surrogate is used to encourage the predicted vessel centerline to overlap with the ground-truth vessel region and to reduce discontinuities in thin branches:

(34) $$L_{\mathrm{topology}}=\sum_{k=0}^{1}W_{2}\;,\left({\mathrm{PH}}_{k}(\hat{Y}),\;\;{\mathrm{PH}}_{k}(Y)\right)$$

This term encourages the model to produce segmentation masks whose topological structures match those of the ground truth, effectively reducing broken vessels and false connections.

#### 3.8.4. Boundary Loss

For applications that demand high boundary fidelity, an optional boundary loss can be added. It measures the spatial discrepancy between the predicted boundary ∂P  and the ground-truth boundary ∂G using a distance transform:

(35) $$L_{\mathrm{boundary}}=\frac{1}{|\partial G|}\sum_{x\in \partial G}d(x,\partial P)$$

where d(x,∂P) is the minimum Euclidean distance from a ground-truth boundary point x  to the predicted boundary. Since vessel boundaries are inherently fractal, we further incorporate a fractal-dimension constraint:

(36) $$L_{\mathrm{boundary}}^{\mathrm{FD}}=L_{\mathrm{boundary}}+\gamma |D_{f}(P)-D_{f}(G)|$$

Here, $D_{f}(\cdot )$ is the fractal dimension of the boundary curve, and γ is a weighting coefficient (set to 0.1 in our experiments). This boundary loss is optional; in our main experiments, we keep it disabled to maintain a lightweight model, but we examine its potential benefit in the ablation study.

#### 3.8.5. Differentiable Implementation and Reproducibility of CFLoss

To improve the reproducibility of CFLoss and clarify how the structural constraints are implemented during network training, this section describes the practical implementation of the fractal constraint, local fractal-distribution alignment, topology-aware surrogate, and Betti error evaluation. In this study, CFLoss is designed as a differentiable structural regularization term that can be optimized through standard back-propagation. Discrete topological measurements, such as the Betti error, are used only for post-training evaluation and are not directly included in the training objective.

For the artery–vein segmentation task, the network outputs artery, vein, and background probability maps. The pixel-level loss is computed for the artery and vein classes to supervise category-specific prediction. In contrast, the structural part of CFLoss is computed on the complete vessel foreground map. Specifically, the predicted artery and vein probability maps are merged into a vessel probability map, and the ground-truth artery and vein masks are also merged into a binary vessel mask. This design is used because fractal complexity, vascular continuity, and topological consistency mainly describe the morphology of the entire vascular tree rather than the semantic category of each vessel pixel.

The fractal-dimension constraint is implemented using a differentiable soft box-counting strategy. Classical box-counting requires binary thresholding and hard counting of occupied boxes, which are not suitable for gradient-based optimization. Therefore, the predicted vessel probability map is used directly without binarization. At each predefined box scale, the image is divided into local boxes, and the occupancy of each box is estimated by a soft probability-based operation. A box with a higher vessel probability contributes more strongly to the box count, whereas a box with a low vessel probability contributes less. In this way, the number of occupied boxes becomes a continuous value rather than a discrete count. The fractal dimension is then estimated from the relationship between box scale and soft box occupancy. Because the whole process is based on continuous probability maps and differentiable operations, gradients can be propagated back to the segmentation network.

To capture local vascular heterogeneity, the global fractal constraint is further complemented by local fractal-distribution alignment. The vessel probability map and the ground-truth vessel mask are divided into local patches using a sliding-window strategy. In our implementation, the patch size is set to 32 × 32 and the stride is set to 8. A soft fractal descriptor is computed for each local patch, producing a set of local fractal values for both the prediction and the ground truth. These local values are then converted into soft histograms rather than hard histograms. The soft histogram assigns each local fractal value to nearby bins in a continuous manner, which keeps the distribution comparison differentiable. The predicted and ground-truth local fractal distributions are aligned using a symmetric distribution-distance loss. This local constraint encourages the model to preserve not only the overall complexity of the vascular tree but also the local structural variations in terminal vessels, bifurcation regions, and low-contrast vessel branches.

The topology-related component of CFLoss is implemented as a differentiable topology-aware surrogate rather than an exact persistent-homology loss. Exact persistent homology and Betti-number computation usually involve thresholding, connected-component analysis, or persistence-diagram matching, which are difficult to integrate directly into ordinary end-to-end network training. To avoid this problem, we use a soft-skeleton-based connectivity constraint during training. The soft skeleton is obtained from the vessel probability map through differentiable morphological operations based on iterative pooling. This soft skeleton approximates the centerline structure of the predicted vessel tree while preserving differentiability. The topology-aware surrogate encourages the predicted vessel skeleton to overlap with the ground-truth vessel region and also encourages the ground-truth skeleton to be covered by the predicted vessel probability map. This bidirectional constraint helps reduce broken vessels, missing thin branches, and structurally implausible discontinuities.

It is important to distinguish the topology-aware surrogate used during training from the Betti error reported in the experiments. The Betti error is not used as a loss function. It is calculated only after inference for evaluation purposes. During evaluation, the predicted probability map is first binarized using a fixed threshold of 0.5. Connected components and holes are then computed on the binary prediction and compared with those of the binary ground-truth mask. The resulting Betti error measures the discrepancy in topological structure between the prediction and the reference annotation. Since this procedure relies on discrete binarization and connected-component counting, it is not differentiable and is therefore excluded from the training objective.

The final CFLoss used in the main experiments consists of the pixel-level segmentation loss, the differentiable fractal constraint, and the soft topology-aware surrogate. The optional boundary loss is disabled in the main experiments to maintain computational efficiency and is considered only in the ablation analysis. The weights of the fractal and topology-aware terms are set to 0.1 and 0.05, respectively. All structural losses are computed on the merged vessel foreground map, whereas the artery–vein category discrimination is supervised by the pixel-level loss. This separation allows the model to learn class-specific artery and vein predictions while still preserving the global morphology and connectivity of the retinal vascular tree.

This implementation ensures that all loss terms used during training are compatible with standard gradient-based optimization. At the same time, discrete topological metrics such as the Betti error are retained as independent evaluation indicators. By separating differentiable structural supervision from non-differentiable topology evaluation, CFLoss can be implemented reproducibly while providing meaningful constraints on vascular complexity and connectivity.

## 4. Experiments and Analysis of Results

### 4.1. Datasets and Data Processing

The DRIVE-AV dataset is a commonly used public dataset for retinal artery–vein classification and vascular structure analysis in fundus images. Unlike binary retinal vessel segmentation datasets that only distinguish vessel pixels from the background, DRIVE-AV provides separate annotations for retinal arteries and veins, making it suitable for evaluating the ability of a model to classify vascular pixels into different vessel categories. Specifically, DRIVE-AV consists of 40 color retinal fundus images, including 20 images for training and 20 images for testing, with each image having a resolution of 565 × 584 pixels. Each image is accompanied by manual artery and vein annotations, which serve as the reference standard for training and evaluation. In this study, the DRIVE-AV dataset was used to evaluate the proposed method for the artery–vein segmentation/classification task. The model predicts artery, vein, and background regions, and the performance is reported not only for the overall vessel region but also separately for arteries and veins.

During training, all images and their corresponding artery–vein annotation masks were resized to 720 × 720 to fit the network input size and available computational resources. The learning rate was set to 0.0008, the batch size to two, and the total number of training epochs to 500. Adam was used as the optimizer. Data augmentation included horizontal and vertical flipping, rotation, and color enhancement. Ten percent of the training images were used as a validation set for learning-rate scheduling and model selection. The learning rate was reduced by half when the training loss did not decrease for 50 consecutive epochs, and training was stopped when the loss did not decrease for 200 consecutive epochs. The model checkpoint with the highest validation F1-score was retained. For class-wise evaluation, artery and vein predictions were evaluated separately against their corresponding ground-truth masks. Overall vessel-level results were obtained by combining artery and vein predictions into a single vessel mask only for auxiliary comparison. A comprehensive geometric and color augmentation pipeline with fixed quantitative hyperparameters was uniformly applied to training samples from both the DRIVE-AV and LES-AV datasets, including random horizontal and vertical flipping, each triggered with an independent probability of 0.5, with segmentation masks undergoing synchronous transformation to maintain pixel-wise label correspondence; random rotation with angles uniformly sampled between −15° and +15°, utilizing zero padding for out-of-bound pixels; bilinear interpolation for fundus images and nearest-neighbor interpolation for binary artery–vein masks to avoid blurred soft labels; and constrained color jitter with independent random perturbations: brightness scaling factors sampled within [0.7, 1.3], contrast adjustment factors ranging from [0.8, 1.2], and gamma correction coefficients uniformly drawn from [0.8, 1.2], with all transformed RGB pixel intensities clamped to the valid range [0, 255] after color manipulation.

The LES-AV dataset is another public retinal fundus image dataset designed for artery–vein segmentation and vascular structure analysis. Compared with DRIVE-AV, LES-AV contains higher-resolution, optic-disk-centered fundus images and provides manual annotations for retinal arteries and veins. Specifically, the dataset consists of 22 color fundus images with a resolution of 1620 × 1444 pixels. Each image is associated with artery and vein labels, making the dataset suitable for assessing the performance of artery–vein classification methods under more challenging imaging conditions. Owing to its higher image resolution and detailed vascular annotations, LES-AV provides a challenging benchmark for evaluating whether the proposed model can distinguish arteries from veins while preserving fine vessels, vascular continuity, and complex branching structures.

In this study, LES-AV was used as an additional benchmark to evaluate the generalization ability of the proposed SDA-SwinNet for the artery–vein segmentation/classification task. The same preprocessing strategy was applied as in the DRIVE-AV experiments. All fundus images and corresponding artery–vein masks were resized to 720 × 720 before being fed into the network. The training settings, including the learning rate, batch size, optimizer, number of epochs, data augmentation strategy, learning-rate scheduling, early stopping criterion, and model selection strategy, were kept consistent with those used for DRIVE-AV. We evaluated both datasets to test generalizability. DRIVE-AV is lower-resolution and more standard; LES-AV is higher-resolution and more challenging. The consistent improvement on both suggests that our method is robust, though the LES-AV results should be interpreted with the resolution trade-off in mind.

### 4.2. Evaluation Metrics

In vessel segmentation and artery–vein segmentation tasks, performance evaluation should consider not only overall prediction accuracy but also the ability to identify fine vascular structures. Therefore, sensitivity (Sen), F1-score (F1), intersection over union (IoU), mean squared error (MSE), and accuracy (ACC) were adopted as evaluation metrics in this study.

Sensitivity measures the model’s ability to identify positive samples (vessel pixels). It is defined as the proportion of true positives (TP) among all actual positives (TP + FN).

(37) $$\mathrm{Sen}=\frac{TP}{TP+FN}$$

Here, TP denotes the number of pixels correctly predicted as vessels, and FN denotes the number of pixels that are actually vessels but are predicted as background.

The F1-score is the harmonic mean of precision and recall (i.e., sensitivity) and provides a comprehensive measure of both.

(38) $$F1=2\;\cdot \;\frac{\mathrm{Precision}\cdot \mathrm{Recall}}{\mathrm{Precision}+\mathrm{Recall}}=\frac{2\cdot TP}{2\cdot TP+FP+FN}$$

IoU (intersection over union) measures the overlap between the prediction and the ground truth and is a commonly used metric in semantic segmentation. Here, FP denotes the number of background pixels that are incorrectly predicted as vessels.

(39) $$\mathrm{IOU}=\frac{TP}{TP+FP+FN}$$

Mean squared error (MSE) measures the average squared difference between predicted and ground-truth pixel values and reflects the overall prediction error.

(40) $$\mathrm{MSE}=\frac{1}{N}\sum_{i=1}^{N}{\left(y_{i}-{\hat{y}}_{i}\right)}^{2}$$

Here, $y_{i}$ denotes the ground-truth pixel value, ${\hat{y}}_{i}$ denotes the predicted value, and N denotes the total number of pixels. Accuracy (ACC) measures the proportion of correctly predicted pixels, including both vessel and background pixels.

(41) $$\mathrm{ACC}=\frac{TP+TN}{TP+TN+FP+FN}$$

The Betti error is introduced to quantify the topological discrepancy between the predicted vessel mask and the ground truth. It measures the difference in the number of connected components (k=0) and holes/loops (k=1) between the two binary images. Let $b_{k}(\hat{Y})$ and $b_{k}(Y)$ denote the -th Betti numbers (i.e., the number of independent k-dimensional topological features) of the prediction and the ground truth, respectively. The Betti error is defined as follows:

(42) $$\mathrm{Bettierror}=\sum_{k=0}^{1}|b_{k}\left(\hat{Y}\right)-b_{k}\left(Y\right)|$$

A lower Betti error indicates better preservation of vascular connectivity and overall topology.

In all experiments, the MSE is computed on binary masks and multiplied by 100 for percentage-style reporting in the tables. Therefore, the MSE values reported in Table 1, Table 2, Table 3, Table 4, Table 5, Table 6 and Table 7 are denoted as MSE (%), and lower values indicate better performance.

### 4.3. Ablation Study

To verify the contribution of each module to overall performance, the improved Shift-ASPP module, the HF-Bridge, and the fractal-dimension-based CFLoss were introduced progressively. As shown in Table 1, the baseline model achieved an F1-score of 69.29%, an IoU of 58.24%, and an accuracy of 94.50%. After introducing only the Shift-ASPP module, the F1-score increased to 71.72%, indicating that zero-parameter receptive-field expansion effectively enhanced the network’s ability to capture fine vessels and terminal structures. After further incorporating the HF-Bridge, the F1-score rose to 72.06%, validating the role of this module in strengthening contextual feature fusion and alleviating the semantic gap. When CFLoss was additionally introduced, the final model achieved Sen, F1, IoU, and ACC scores of 77.21%, 73.13%, 63.06%, and 95.34%, respectively, on the DRIVE-AV dataset, showing consistent improvements over the baseline across all metrics.

**Table 1:** Ablation analysis of different modules.

Baseline	Shiftaspp	HF	CFLoss [23]	Sen	F1	IOU	MSE (%)	ACC
√				73.7	69.29	58.24	5.23	94.50
√	√			73.85	71.72	61.98	4.85	95.22
√		√		74.76	72.06	60.67	5.00	95.02
√			√	76.89	73.01	62.79	4.78	95.21
√	√	√	√	77.21	73.13	63.06	4.77	95.34

*“CFLoss” refers to the fractal-constrained loss adopted from [23]. All experiments use Swin-UNet as the backbone. Results are reported on the DRIVE-AV dataset.*

Unless otherwise specified, artery and vein results are computed class-wise by treating the corresponding vessel category as the positive class. The overall vessel-level result is obtained by merging artery and vein predictions into a single foreground vessel mask.

### 4.4. Comparative Experiments

Table 2, Table 3 and Table 4 summarize the results on DRIVE-AV. To isolate the contribution of the loss function, we first trained the standard Swin-UNet backbone with CF-Loss [23] (denoted as ‘Baseline + CF-Loss’ in Table 2). This baseline achieves an F1-score of 73.01% and a Betti error of 7.98. Our full SDA-SwinNet, which adds Shift-ASPP and HF-Bridge to the same backbone and loss, achieves a slightly higher F1-score (73.13%) and a notably lower Betti error (7.01). The gap in F1 is modest, but the Betti error reduction suggests that architectural enhancements help preserve vascular topology beyond what the loss function alone can achieve. On artery and vein subsets (Table 3 and Table 4), SDA-SwinNet shows consistent gains over AC [24], GC [25], and clDice [22], though the improvements are not uniform across all metrics.

**Table 2:** Comparative trial on DRIVE-AV.

				DRIVE-AV			
Model	Backbone	Loss Function	Sen ↑	F1 ↑	IOU ↑	MSE (%)	Betti Error ↓
AC [24]	U-Net	Active Contour	65.03	68.10	53.12	3.12	10.76
GC [25]	U-Net	Graph Cuts	64.10	69.53	54.05	3.07	25.98
clDice [24]	U-Net	clDice	67.68	70.15	56.69	3.05	10.02
CF-Loss [23]	Swin-UNet	CF-Loss [23]	70.01	73.01	59.34	**2.97**	7.98
SDA-SwinNet **(Ours)**	Swin-UNet + Ours	CF-Loss [23] + Topology Surrogate	**72.37**	**73.13**	**60.98**	3.02	**7.01**

*“CF-Loss [23]” denotes using the loss function proposed in [23] with the standard Swin-UNet backbone. “SDA-SwinNet” incorporates our proposed Shift-ASPP and HF-Bridge modules on top of Swin-UNet, trained with the same CF-Loss, plus an additional topology-aware surrogate.*

**Table 3:** Comparative trial on DRIVE-AV (vein).

		DRIVE-AV (Vein)			
Model	Sen ↑	F1 ↑	IOU ↑	MSE (%)	Betti Error ↓
AC [24]	72.13	72.02	56.19	3.34	13.26
GC [25]	72.94	72.73	57.63	3.54	26.07
clDice [22]	73.78	73.84	60.13	3.41	13.22
CF-Loss [23]	77.01	77.01	63.09	**2.78**	10.11
SDA-SwinNet **(Ours)**	**78.37**	**78.73**	**65.18**	2.96	**9.98**

*“Swin-UNet + CF-Loss” denotes the standard Swin-UNet backbone trained with the loss function from [23]. “SDA-SwinNet” adds our proposed Shift-ASPP and HF-Bridge modules. Best results are shown in bold.*

**Table 4:** Comparative trial on DRIVE-AV (artery).

		DRIVE-AV (Artery)			
Model	Sen ↑	F1 ↑	IOU ↑	MSE (%)	Betti Error ↓
AC [24]	65.53	67.12	52.09	3.06	8.76
GC [25]	65.74	68.63	52.53	3.15	16.57
clDice [22]	66.12	71.34	53.34	2.81	7.78
CF-Loss [23]	**70.21**	73.03	56.36	2.71	5.11
SDA-SwinNet **(Ours)**	70.17	**75.23**	**57.89**	**2.56**	**4.99**

*For table footnotes, please refer to the notes under Table 2.*

In contrast, traditional models such as AC, GC, and clDice demonstrate weaker performance across multiple metrics. The AC model achieves an F1-score of 68.10 and an IOU of 53.12, indicating significant shortcomings in segmentation accuracy—particularly in detail restoration and topological consistency—with a Betti error of 10.76, suggesting incomplete vascular structures. While GC and clDice outperform AC on certain metrics, they still fall short of the precision achieved by the CF-Loss and our full SDA-SwinNet, especially in handling complex vascular regions, where their topological consistency and detail restoration capabilities are limited. Overall, SDA-SwinNet achieves competitive performance on both datasets. The improvement over CF-Loss [23] is modest (73.13 vs. 73.01 in F1), but the reduction in the Betti error suggests better topological preservation. Whether this translates to clinically meaningful gains requires further investigation.

Table 5, Table 6 and Table 7 show the results on LES-AV. The overall F1-score of SDA-SwinNet is 67.85%, slightly higher than the Swin-UNet + CF-Loss baseline (66.87%). The Betti error drops from 6.58 to 5.61, echoing the DRIVE-AV finding that architectural improvements contribute to topological consistency. However, the gains on LES-AV are more modest than those on DRIVE-AV—likely due to the resolution trade-off discussed earlier (Section 5.2).

**Table 5:** Comparative trial on LES-AV (mean value).

		LES-AV			
Model	Sen↑	F1 ↑	IOU ↑	MSE (%)	Betti Error ↓
AC [24]	60.01	64.56	47.34	3.02	9.46
GC [25]	62.10	63.93	48.11	2.97	15.23
clDice [22]	62.98	65.43	50.39	2.76	10.12
CF-Loss [23]	72.11	66.87	52.34	**2.57**	6.58
SDA-SwinNet **(Ours)**	**72.57**	**67.85**	**53.78**	2.68	**5.61**

*For table footnotes, please refer to the notes under Table 2.*

**Table 6:** Comparative trial on LES-AV (vein).

		LES-AV (Vein)			
Model	Sen ↑	F1 ↑	IOU ↑	MSE (%)	Betti Error ↓
AC [24]	60.21	63.34	49.54	3.13	13.56
GC [25]	62.09	64.10	50.21	3.04	25.76
clDice [22]	62.14	65.56	51.01	3.18	15.02
CF-Loss [23]	64.45	**68.97**	53.54	2.74	7.88
SDA-SwinNet **(Ours)**	**66.42**	67.98	**55.68**	**2.66**	**6.76**

*For table footnotes, please refer to the notes under Table 2.*

**Table 7:** Comparative trial on LES-AV (artery).

		LES-AV (Artery)			
Model	Sen ↑	F1 ↑	IOU ↑	MSE (%)	Betti Error ↓
AC [24]	60.02	61.45	47.21	2.87	7.86
GC [25]	61.21	62.03	46.81	2.63	7.64
clDice [22]	61.83	63.11	47.11	2.71	7.22
CF-Loss [23]	62.15	64.32	48.89	**2.56**	4.58
SDA-SwinNet **(Ours)**	**64.26**	**65.01**	**50.01**	2.60	**5.06**

For vein and artery subsets (Table 6 and Table 7), the pattern is mixed. SDA-SwinNet outperforms the baseline on artery segmentation (F1: 65.01 vs. 64.32) but slightly underperforms on vein segmentation (F1: 67.98 vs. 68.97). This suggests that the proposed modules may not benefit all vessel categories equally. Traditional methods, AC [24], GC [25], and clDice [22], consistently lag behind both CF-Loss-based configurations across most metrics.

Overall, SDA-SwinNet achieves competitive performance on LES-AV, but the improvements are less pronounced than on DRIVE-AV. Whether this reflects inherent dataset difficulty, a resolution mismatch, or a limitation of the architecture itself remains unclear and warrants further investigation.

### 4.5. Parameterized Experiment

The baseline model has 42.5 million parameters, 38.2 G FLOPs, and an inference speed of 23.8 FPS, which falls within a reasonable range for a Swin-UNet-like architecture. After incorporating HF-Bridge, the parameter count increases to 48.9 million, FLOPs rise to 42.7 G, and FPS drops to 21.9. This indicates that HF-Bridge is the primary module contributing to increased complexity, as it requires multi-layer feature alignment, cross-scale fusion, and Res2NetBlock-based multi-scale convolutional enhancement—resulting in significantly higher parameter and computational demands compared to Shift-ASPP. When CFLoss is added alone, its parameter count, FLOPs, and FPS remain identical to those of the baseline.

The complete SDA-SwinNet model has 49.2 million parameters, 42.8 G FLOPs, and achieves 21.9 FPS. These results align with the complexity trends of its components: Shift-ASPP introduces minimal overhead and HF-Bridge accounts for the primary overhead, while CFLoss has no impact on inference complexity. Overall, although the full model requires more computation than the baseline, its inference speed only drops from 23.8 FPS to 21.9 FPS, maintaining a highly practical inference efficiency.

### 4.6. In-Depth Discussion of Atypical Experimental Observations

Combining the ablation experiments, category-wise comparison results and complexity analysis above, three counterintuitive experimental phenomena are consistently observed across all test benchmarks: (1) HF-Bridge yields substantially larger performance gains for arterial segmentation than venous segmentation; (2) the performance improvements brought by Shift-ASPP and HF-Bridge are markedly weaker on the LES-AV dataset compared with DRIVE-AV; and (3) the reduction in Betti error is far more prominent than the marginal increment in the F1-score. This subsection elaborates on the intrinsic mechanisms behind these phenomena, considering anatomical priors, dataset characteristics, network structural design and the mathematical properties of evaluation metrics.

#### 4.6.1. Disparate Performance Boosts of HF-Bridge on Arterial and Venous Segmentation

The consistent discrepancy in module-induced gains between arteries and veins originates from two coupled factors: inherent morphological differences between retinal arteries and veins and the structural bias of the proposed HF-Bridge module toward tiny tubular structures.

First, retinal arteries and veins exhibit distinct anatomical characteristics in fundus images. Retinal arteries are generally thinner, with denser hierarchical bifurcations and numerous delicate terminal capillaries. Most arterial branches are distributed in peripheral, low-contrast regions adjacent to the image background, making them prone to incomplete segmentation and structural fragmentation under standard encoder–decoder architectures with naive skip connections. In contrast, retinal veins possess wider lumens, fewer fine peripheral branches, and more continuous main trunks with higher pixel occupancy. The baseline Swin-UNet already achieves satisfactory segmentation for thick venous regions, leaving limited room for further performance optimization.

Second, the core functionality of HF-Bridge lies in multi-stage dense cross-scale feature aggregation empowered by Res2Net blocks. The module progressively upsamples deep semantic feature maps and aligns them with high-resolution shallow texture features, which specifically compensates for lost microvascular details during decoder upsampling. This hierarchical fusion pipeline delivers the most significant regularization effect on sparse, fragmented arterial micro-branches. For thick, continuous venous regions, however, the multi-scale detail enhancement mechanism of HF-Bridge cannot generate equivalent performance increments, as venous segmentation suffers far less from feature loss and structural discontinuity.

Third, severe class imbalance amplifies the performance gap. Across both DRIVE-AV and LES-AV, the total pixel count of arterial vessels is significantly lower than that of veins, rendering arteries a hard minority class. The baseline model exhibits severe under-segmentation of arterial terminals, while venous segmentation already reaches a relatively saturated performance ceiling. Consequently, HF-Bridge’s complementary feature fusion produces a numerically larger improvement in arterial evaluation metrics.

#### 4.6.2. Diminished Performance Improvements on the LES-AV Dataset

The limited gain of SDA-SwinNet on LES-AV arises from irreversible information loss caused by unified preprocessing, intrinsic dataset distribution differences, and inconsistent vascular complexity between the two benchmarks.

1. Resolution mismatch and destructive downsampling preprocessing

All input images are uniformly resized to 720 × 720 before network feeding. The native resolution of DRIVE-AV is 565 × 584; upsampling to 720 × 720 only introduces minor interpolation without erasing fine vascular textures. In contrast, LES-AV raw images are captured at 1620 × 1444, and aggressive downsampling smooths out tiny terminal capillaries and subtle bifurcation structures. The core advantages of Shift-ASPP and HF-Bridge rely on intact fine-scale vascular textures to expand receptive fields and reconstruct fragmented branches. Once microvascular information is eliminated by compression, the optimization targets of the two proposed modules largely disappear, drastically weakening their marginal performance contributions.

2. Vascular distribution discrepancies between datasets

DRIVE-AV covers a complete fundus field of view, containing a balanced mixture of thick main trunks and distal fine branches, which fully activates the multi-scale modeling capacity of Shift-ASPP and the cross-layer compensation of HF-Bridge. LES-AV images are cropped around the optic disk and are dominated by thick central vessels with scarce peripheral microvasculature. The lack of multi-scale vascular hierarchies restricts the scenario scope where the proposed modules can deliver observable improvements.

3. Imaging noise and contrast heterogeneity

Native high-resolution LES-AV images contain complex artifacts including fundus specular reflections and pathological interferences. After downsampling, noise signals overlap with faint microvascular textures, reducing the feature discriminability between vessels and the background. DRIVE-AV features uniform illumination and low background noise, allowing the channel-wise spatial shift operation in Shift-ASPP to effectively disentangle vascular structures from background clutter and produce measurable performance gains.

#### 4.6.3. Mismatched Magnitudes of Betti Error Reduction and F1-Score Improvement

A consistent observation across all comparative experiments is that the topological metric Betti error undergoes a substantial drop, while the pixel-level F1-score only achieves marginal growth. This mismatch stems from fundamental differences in evaluation dimensions, the optimization priorities of the proposed modules, and the numerical saturation characteristics of the two metrics.

F1-score is a region-wise pixel-matching metric that quantifies the overlap between predicted and ground-truth vessel masks. It remains robust against minor structural defects such as sparse tiny breaks or micro-holes, provided the overall foreground pixel coverage is well preserved. In contrast, the Betti error quantifies topological inconsistency by summing absolute differences in Betti numbers (connected components for k = 0, closed loops for k = 1) between prediction and ground truth. It is extremely sensitive to fragmented vessel segments, spurious branches, and tiny cavities. The baseline Swin-UNet + CF-Loss already achieves high pixel-wise overlap, saturating the F1-score with limited room for further promotion; meanwhile, numerous discontinuous fine branches and redundant topological defects yield a high initial Betti error, leaving ample optimization space for structural regularization.

The two proposed architectural modules are deliberately designed to mitigate topological defects rather than boost global pixel-matching ratios. Shift-ASPP expands the effective receptive field via cost-free spatial shifting, enabling continuous sampling of elongated vascular textures to truncate broken terminals. HF-Bridge eliminates the semantic gap between shallow and deep features via dense cross-layer fusion, recovering disconnected micro-branches during upsampling. Neither module drastically modifies the total proportion of correctly predicted vessel pixels, so the F1-score exhibits trivial increments. Nevertheless, both modules directly reduce disconnected components and spurious holes, which yields a prominent decline in the Betti error.

The pixel-wise loss term Lpixel dominates the back-propagation gradient, while the fractal constraint and topology-aware surrogate act as lightweight regularizers with small weighting coefficients. The baseline model converges to a near-optimal local minimum for pixel matching, which restricts further F1-score growth. The enhanced multi-scale features extracted by Shift-ASPP and cross-layer complementary features from HF-Bridge provide superior input representations for the structural regularization terms, substantially minimizing fractal and topology loss penalties. The network prioritizes optimizing vascular connectivity under structural constraints, leading to disproportionate improvements in topological metrics.

The baseline overall F1-score on DRIVE-AV reaches 73.01%, which lies in a high-value saturated interval where subtle pixel corrections only generate percentage-level marginal gains. The baseline Betti error equals 7.98, with a wide numerical range; repairing a small number of broken branches and cavities directly reduces the value by integer magnitudes, resulting in a visually significant relative decline.

## 5. Conclusions and Limitations

### 5.1. Conclusions

To address the complexity of retinal vascular structures, the difficulty of identifying fine vessels, and the limited feature extraction and global modeling capabilities of conventional methods, this paper proposes a fundus retinal vessel segmentation network based on Swin-UNet. While maintaining high segmentation accuracy, the model effectively reduces model complexity and is therefore more suitable for practical scenarios such as clinical decision support.

First, at the backbone level, the Swin-UNet architecture is redesigned by introducing an efficient Transformer-based encoding and decoding framework to enhance the global modeling capability. On this basis, an improved Shift-ASPP module is proposed to realize zero-parameter receptive-field expansion through channel-wise spatial shift, effectively improving the capture of multi-scale vascular features. In addition, a multi-fusion HF-Bridge is designed to strengthen inter-layer interaction and fusion of features, thereby compensating for the deficiencies of conventional structures in semantic transmission and detail preservation. Finally, we adopt the fractal-constrained CF-Loss from [23] as a structural regularizer to guide the model toward preserving vascular morphology and topological consistency. In combination with our proposed Shift-ASPP and HF-Bridge modules, this loss contributes to improved segmentation of fine vessels and boundary regions.

Experimental results on DRIVE-AV show clear improvements. On LES-AV, the gains are more modest—likely due to our downsampling preprocessing. Patch-based training at a native resolution remains an open direction for future work. In particular, the model exhibits higher continuity and boundary fidelity in microvascular regions and artery–vein crossing areas. The ablation study confirms that each module contributes positively. The improvements are consistent across both datasets.

Overall, SDA-SwinNet maintains competitive segmentation accuracy with a reasonable computational cost, providing a scalable solution for retinal vessel segmentation. Future work will further explore its generalization ability across devices, populations, and multimodal medical images, and will combine few-shot learning and knowledge distillation to develop more intelligent clinical decision-support systems.

### 5.2. Limitations

Although the proposed SDA-SwinNet achieves promising performance on retinal vessel segmentation and artery–vein segmentation tasks, several limitations remain.

First, the experimental validation was mainly conducted on relatively small public datasets, including DRIVE-AV and LES-AV. These datasets contain a limited number of annotated fundus images, which may restrict the statistical reliability of the reported results. Although data augmentation was adopted during training, the diversity of imaging devices, ethnic populations, pathological conditions, and image quality variations was still insufficient. Therefore, further validation on larger multi-center datasets is required to fully evaluate the generalization ability of the proposed method.

Second, all input images were resized to a fixed resolution of 720 × 720 before being fed into the network. While this preprocessing strategy simplifies training and reduces computational cost, it may alter the original aspect ratio or fine vessel morphology, especially for high-resolution images such as those in the LES-AV dataset. Since retinal vessels contain many thin and tortuous structures, resolution changes may affect the representation of terminal vessels, vessel width, and local topological characteristics. Future work should investigate patch-based training, multi-resolution inference, or aspect-ratio-preserving preprocessing strategies to better preserve original vascular details.

Third, the proposed CFLoss introduces fractal-dimension and topology-aware constraints to improve structural consistency. However, the computation of fractal dimension, local fractal descriptors, and topological properties may introduce additional computational overhead during training. Moreover, some operations related to fractal analysis and persistent homology are not naturally differentiable and require suitable approximation strategies. Although the proposed formulation provides structural guidance for vessel segmentation, further work is needed to develop more efficient and fully differentiable implementations.

Fourth, the current study mainly focuses on segmentation accuracy and structural consistency, while the clinical interpretability of the segmentation results has not been fully explored. Retinal vascular features such as the fractal dimension, vessel density, tortuosity, and artery–vein ratio are clinically meaningful biomarkers. However, this work does not further analyze how improvements in segmentation quality affect the reliability of downstream clinical feature measurements. Future studies should evaluate whether the proposed method can improve disease risk assessment and clinical biomarker extraction.

Fifth, the comparative experiments were limited to selected baseline methods and loss functions. Although the proposed model demonstrates advantages over the compared methods, more comprehensive comparisons with recent Transformer-based segmentation networks, lightweight medical segmentation models, and state-of-the-art artery–vein segmentation methods are still necessary. In addition, statistical significance testing and cross-dataset validation should be included in future work to provide stronger evidence for the effectiveness and robustness of the proposed approach.

Finally, although we provide a basic complexity analysis in Table 8, a more comprehensive evaluation is still needed. In particular, memory consumption on different hardware platforms, inference time on low-resource devices, and comparisons with other lightweight segmentation models should be examined. These factors are critical for real-world clinical deployment. Future work will explore model compression strategies such as knowledge distillation, pruning, and lightweight attention mechanisms to further improve efficiency.

## Figures and Tables

**Table 8:** Complexity analysis of different modules in SDA-SwinNet.

Baseline	Shiftaspp	HF	CFLoss	Param (M)	FLOPs (G)	FPS
√				42.5	38.2	23.8
	√			42.8	38.3	23.5
		√		48.9	42.7	21.9
			√	42.5	38.2	23.8
	√	√	√	49.2	42.8	21.9

## Data Availability

The data used in this study are publicly available.
